# Anticoagulation With Platelet Transfusions for Acute Limb Ischemia With Severe Thrombocytopenia

**DOI:** 10.7759/cureus.41255

**Published:** 2023-07-01

**Authors:** Khalid Shakfeh, Damian A Laber

**Affiliations:** 1 Internal Medicine, University of South Florida Morsani College of Medicine, Tampa, USA; 2 Internal Medicine, Tampa General Hospital, Tampa, USA; 3 Satellite and Community Oncology, Moffitt Cancer Center, Tampa, USA

**Keywords:** systemic anticoagulation, mycosis fungoidis, protein calories malnutrition, thrombocytopenia, acute limb ischaemia

## Abstract

Acute limb ischemia (ALI) is a medical and surgical emergency, and the mainstays of treatment are therapeutic anticoagulation and surgery. These interventions require adequate platelet count and functionality. Anticoagulation and surgery can be complicated in thrombocytopenic patients and require interdisciplinary management for optimal outcomes, as literature is limited in this population. We present a case of a patient with severe thrombocytopenia who developed limb ischemia from cancer-associated thrombosis (CAT). We propose a management strategy for anticoagulation and perioperative platelet transfusion, with successful revascularization without adverse bleeding events. While successful, more data is required to investigate long-term outcomes.

## Introduction

The most common causes of acute limb ischemia (ALI) are typically divided into embolic and thrombotic events. Thrombotic events are mostly found in patients with peripheral vascular disease. Common risk factors include uncontrolled hypertension, diabetes, and smoking. Conversely, embolic events are more frequently found in arrhythmia, mostly atrial fibrillation [1]. Regardless, the standard management for both remains the same. ALI with Rutherford grades I-IIB is typically managed by therapeutic anticoagulation, most frequently a heparin drip, followed by surgical intervention [2].

As of the writing of this report, we found no objective published data on anticoagulation for ALI with severe thrombocytopenia. Here, we report the management of a patient who developed ALI treated with anticoagulation and platelet transfusions while his platelet count was less than 30,000 x10^9^/L.

## Case presentation

**Figure 1:**
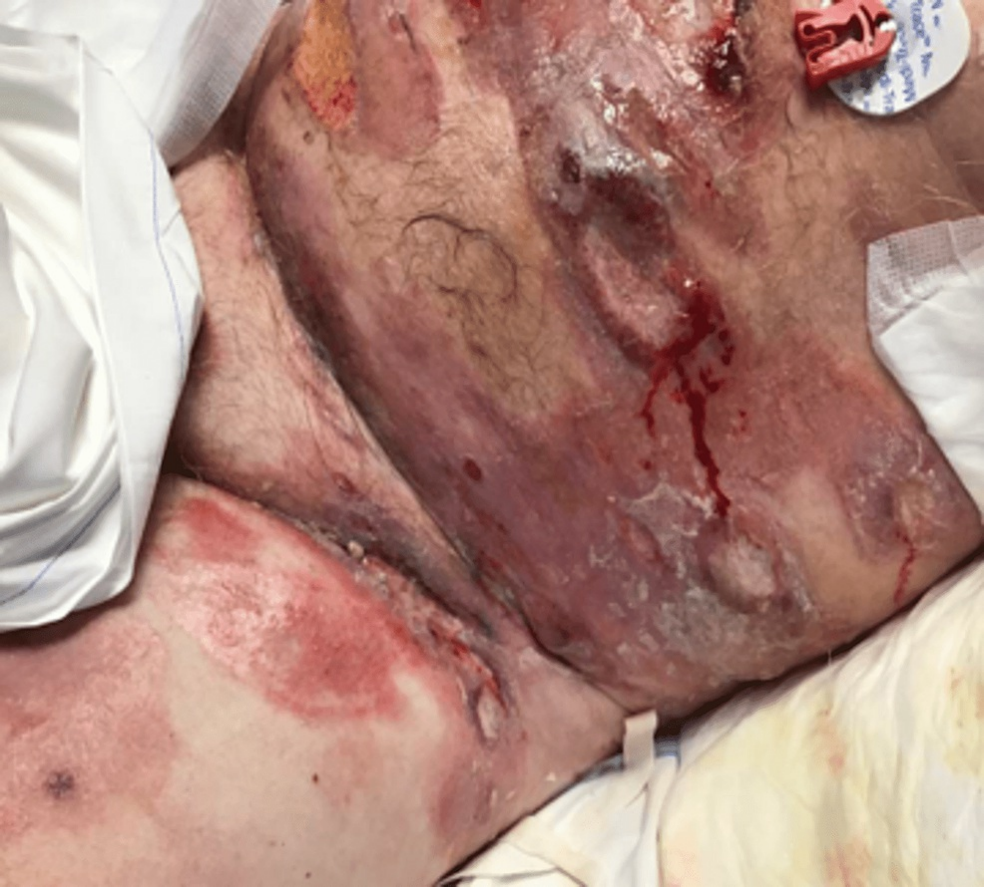
Representative photo of the severity of mycosis fungoides in our patient

A 65-year-old gentleman with refractory cutaneous T-cell lymphoma (mycosis fungoides subtype) presented to the emergency department for worsening oozing from his wounds, with associated pain for several days (Figure 1). The patient had been recently admitted to a local hospital with similar complaints. At that facility, he was found to have *Klebsiella bacteremia* and was hospitalized and treated with IV cefazolin for two weeks. Upon presentation, he was hypotensive, tachycardic, and with approximately 67% of his body covered in wounds associated with his lymphoma. Laboratory tests revealed anemia and thrombocytopenia with a platelet count of 33,000 x10^9^/L (Table 1). Sepsis protocol was initiated, and after fluid resuscitation, his vitals improved.

**Table 1:** Admission complete blood count

	Admission values	Reference range (male)
WBC (×10^9^/L)	5.54	3.8-10.4
Hemoglobin (g/dL)	7.9	13.6-16.9
Hematocrit (%)	23.7	40-50
Platelets (x10^9^/L)	33	153-361

Repeat laboratory values demonstrated a thrombocytopenia of 18,000 x10^9^/L. Throughout the remainder of his hospitalization, his platelets remained around 18-27,000 x10^9^/L. On HD 7, he developed unilateral leg poikilothermia, pulselessness at his dorsalis pedis and posterior tibial arteries, and worsening sensorimotor changes, consistent with Rutherford IIB ALI. He had a biphasic popliteal artery pulse with absent distal pulses. He was transfused four units of platelets until his platelet count was greater than 50,000 x10^9^/L and started on a heparin drip. He was taken to the operating room, where a small clot from his anterior tibial artery was removed. Given his high risk of developing compartment syndrome, fasciotomies were performed prophylactically.

He was thereafter transferred to the intensive care unit for hourly neurovascular checks. He remained on the heparin drip, and his platelets postoperatively were 45-50,000 x10^9^/L. Given planned returns to the operating room for his fasciotomy revisions, he was transfused for a platelet goal of greater than 50,000 x10^9^/L while on the heparin drip. On HD 11/POD 4, he had progressive ischemic changes with signal loss and was taken back to the operating room for a repeat thrombectomy, bovine patch angioplasty, and common iliac artery stenting. Postoperatively, his dorsalis pedis signals remained biphasic.

A thromboembolic workup, including transthoracic echocardiogram, protein C and S levels, factor V, antiphospholipid, cardiolipin, fibrinogen, partial thromboplastin time, prothrombin time, platelet factor 4 antibodies, flow cytometry, and haptoglobin were all normal. A bone marrow aspiration and biopsy one month prior to his presentation demonstrated 20-30% cellularity, without bone marrow involvement from his malignancy. His thrombocytopenia was deemed likely either due to his active malignancy or acute illness.

The platelet goal was decreased to >30,000 x10^9^/L with platelet transfusion support while on a heparin drip. He ultimately received a total of nine units of platelets. He had no significant bleeding complications or ischemic complications.

Unfortunately, due to his progressive malignancy, he decided to transition to comfort measures only and died on HD 17.

## Discussion

There is little data on systemic anticoagulation in patients with ALI and profound thrombocytopenia. Most published studies have revolved around the management of cancer-associated thrombosis (CAT) in patients with thrombocytopenia, mostly venous thromboembolic disease [3], where it is common practice to implement full anticoagulation with platelets greater than 50,000 x10^9^/L, halved between 30,000 x109/L and 50,000 x109/L, and held less than 30,000 x10^9^/L [4]. Anticoagulation is held for platelets less than 30,000 x10^9^/L for concerns of life-threatening bleeding; however, thrombocytopenia is not seen as protective against emboli or thrombi [5]. Another strategy commonly used in CAT during the first 30 days (highest risk for recurrent venous thromboembolism) is full-dose therapeutic anticoagulation with supportive platelet transfusions to maintain platelet count >40-50 × 10^3^/µL [6-7].

In our patient with ALI after the initial thrombectomy, we opted for anticoagulation with supportive platelet transfusions to maintain an initial platelet goal of greater than 50,000 x10^9^/L. With this strategy, he had no bleeding complications but developed re-thrombosis four days later necessitating a repeat thrombectomy. After the second thrombectomy, he was maintained on therapeutic anticoagulation with supportive platelet transfusions, but the platelet goal was decreased to greater than 30,000 x10^9^/L. With this strategy, he had no bleeding complications and no re-thrombosis.

## Conclusions

We acknowledge that this is a case report with very limited data that might not be reproducible since each patient is unique. We hope our experience can be informative for treating physicians encountering similar problems in their practice. Anticoagulation in thrombocytopenic patients with ALI is a very tricky matter. A balance must be obtained between decreasing complications related to clots and the risks of bleeding. We hope our case might stimulate urgently needed studies in the management of ALI with severe thrombocytopenia.
